# Progestin-primed ovarian stimulation with letrozole using different doses of medroxyprogesterone acetate per day: a retrospective cohort study

**DOI:** 10.3389/fendo.2024.1429338

**Published:** 2024-07-12

**Authors:** Hai-long Li, Bei-bei Shen, Zheng-liang He, Hai-li Wang, Zhi-feng Sun

**Affiliations:** ^1^ Reproductive Medicine Center, Renmin Hospital, Hubei University of Medicine, Shiyan, Hubei, China; ^2^ Hubei Key Laboratory of Embryonic Stem Cell Research, Shiyan, China; ^3^ Biomedical Engineering College, Hubei University of Medicine, Shiyan, China; ^4^ The Third Medical School, Hubei University of Medicine, Shiyan, China; ^5^ Hubei Clinical Research Center For Reproductive Medicine, Shiyan, Hubei, China

**Keywords:** medroxyprogesterone acetate, letrozole, controlled ovarian stimulation, PPOS, ClbR

## Abstract

**Background:**

In the progestin-primed ovarian stimulation protocol, the oral administration of medroxyprogesterone acetate has been observed to effectively inhibit the LH surge during ovarian stimulation in patients experiencing infertility. Nevertheless, the use of utilizing medroxyprogesterone acetate during ovarian stimulation can result in more pronounced pituitary suppression, potentially necessitating increased doses of gonadotropins and extended treatment durations. Therefore, it is necessary to determine the optimal dose of medroxyprogesterone acetate, aiming to use relatively lower concentrations of medroxyprogesterone acetate to effectively and safely suppress early LH surges.

**Method:**

This retrospective cohort study included 710 patients who underwent cycles of *in vitro* fertilization or intracytoplasmic sperm injection and were subjected the progestin-primed ovarian stimulation protocol utilizing letrozole between from 1st January 2021 to 31st December 2021. The study population was divided into low, medium, and high concentration groups based on the daily dosage of medroxyprogesterone acetate.The primary focus of this investigation was on the cumulative live birth rate. Secondary outcomes encompassed the occurrence of a premature surge in luteinizing hormone, the quantity of retrieved oocytes, viable embryos, and high-quality embryos, as well as clinical pregnancy rate, abortion rate, ectopic pregnancy rate, and multiple pregnancy rate.

**Results:**

In this study, significant differences were observed among three groups in various parameters including body mass index, baseline levels of Anti-Müllerian hormone and luteinizing hormone, antral follicle count, total dose of gonadotropin, and duration of gonadotropin administration (p<0.05). The number of oocytes and viable embryos were significantly higher in medium group and higher than those in the low dose group. Following adjustments for confounding factors related to medroxyprogesterone acetate for various outcome measures, we conducted multiple regression analysis to investigate the independent effects of daily medroxyprogesterone acetate dosage within the combined progestin-primed ovarian stimulation and letrozole protocol. Following multivariable regression analysis, no disparities were found in embryo characteristics (number of oocytes retrieved, number of available embryos, number of high-quality embryos) or pregnancy outcomes (clinical pregnancy rate, cumulative live birth rate) among the three groups.

**Conclusion:**

Progestin-primed ovarian stimulation with letrozole using different dose of medroxyprogesterone acetate per day was comparable in terms of the number of oocytes retrieved, the number of high-quality embryos, clinical pregnancy rate and cumulative live birth rate after frozen embryo transfer.

## Introduction

The PPOS protocol, introduced by Professor Kuang in 2015, entails pituitary suppression using oral progestins during the follicular phase until the ovulation trigger, rather than employing GnRH analogues. This protocol effectively prevents the oestradiol (E_2_)-induced LH surge by administering MPA and human menopausal gonadotropin (hMG) from the early follicular phase (1). Consequently, the PPOS protocol provides an alternative approach to conventional GnRH analogues (2–5). Oral administration of MPA suppresses the pituitary LH surge in infertile patients during ovarian stimulation (6–8). Due to its detrimental effects on the endometrium, MPA inhibition is paired with a freeze-all strategy (2).

A previous study demonstrated that in older patients with diminished ovarian reserve, the PPOS protocol can improve control over premature LH surges and yield reproductive outcomes similar to those of mild stimulation protocols. Therefore, the PPOS protocol is a feasible alternative to mild stimulation protocols (9). Furthermore, certain studies have suggested that the PPOS protocol is more effective at controlling premature LH rises compared to GnRH antagonists in patients with normal ovarian response (10), poor responders (11) and polycystic ovary responders (12). A recent systematic review indicated that patients using the PPOS protocol exhibit ovarian response characteristics, pregnancy outcomes per transfer, and embryo euploidy rates similar to those observed with GnRH analogs (13). However, because MPA may result in greater pituitary suppression during ovarian stimulation, the PPOS protocol potentially requires higher doses of gonadotropins and longer stimulation durations compared to traditional ovarian stimulation protocols (1), thereby increasing both the economic and time costs for patients. Hence, it is desirable to identify an optimal dose of MPA, aiming to improve its cost-effectiveness, efficiency, and safety.

According to reports, the daily administration of 10 mg of MPA during natural and ovarian stimulation cycles has been reported to effectively suppress spontaneous ovulation (1) (14). Additionally, a study has shown that a daily dosage of 4 mg of MPA in women undergoing controlled ovarian hyperstimulation achieves comparable pregnancy outcomes and endocrine characteristics to those taking 10 mg of MPA daily (15). However, the results obtained in a study suggest 5 mg MPA is not sufficient to inhibit ovulation in all women (14). As of now, it remains unclear whether there is an optimal dose of MPA that can guarantee favorable pregnancy outcomes while effectively preventing spontaneous LH surges. Once the optimal range of MPA concentration is determined, achieving favorable pregnancy outcomes with a relatively lower concentration of MPA, which is more easily attainable, becomes feasible. Our objective is to investigate whether patients receiving varying doses of MPA in the PPOS regimen demonstrate different effects. Therefore, we designed a retrospective cohort study to compare the endocrine characteristics and clinical outcomes of patients undergoing IVF/ICSI treatment with different doses of MPA in the PPOS protocol.

If substantiated, these findings could pave the way for the creation of stimulation regimens that are more convenient and user-friendly, given that MPA is administered orally. This could also result in reduced stimulation costs of time, thereby enhancing patients’ accessibility to assisted reproduction treatments.

## Materials and methods

### Participant

This retrospective data analysis included 710 patients who used letrozole combined with the PPOS regimen and underwent their first IVF/ICSI cycle between from 1st January 2021 to 31st December 2021. This study protocol was formulated in accordance with the requirements of the Declaration of Helsinki of the World Medical Association. And informed consent forms were provided by all patients after they received counseling on infertility treatment and the routine ART process. Patients who intended to undergo ovarian stimulation for preimplantation genetic diagnosis or screening, oocyte donation, and those with severe oligozoospermia, mild oligozoospermia, or azoospermia were excluded from the study. Additionally, women who experienced cycle cancellation or had zero oocytes retrieved were also excluded.

### Controlled ovarian stimulation protocols and laboratory procedure

Letrozole co-treatment within the PPOS protocol (16): within the PPOS protocol, letrozole(Jiangsu Furui Pharmaceutical Trading Co., China) was co-administered at a dose of 2.5 mg/day, starting on days 2–3 of the menstrual cycle. This coincided with the initiation of ovulation induction therapy, which involved intramuscular injections of 75–150 IU/day of gonadotropin. Concurrently, oral administration of 10 mg/day medroxyprogesterone acetate (MPA, Zhejiang Xianju Pharmaceutical Co., China) began before estrogen levels rose. The MPA dosage was gradually adjusted according to serum LH levels until the day of human chorionic gonadotropin (HCG, Lizhu Pharmaceutical Trading Co., China) injection. Meanwhile, ovulation induction involved intramuscular injections of 150–225 IU/day of gonadotropin (urinary gonadotropin, Lizhu Pharmaceutical Trading Co., China; Gonal-F, Merck Serono, Germany), with dosage adjustments based on follicle size and sex hormone levels. When the mean diameter of the dominant follicle reached 20 mm, 0.1 mg of GnRH-a (Decapeptyl; Ferring Pharmaceuticals, Germany) was administered along with a 2000 IU HCG trigger to induce oocyte maturation. Laboratory procedures included oocyte retrieval performed 34–36 hours post-trigger under transvaginal ultrasound guidance using a double lumen aspiration needle. Oocytes were fertilized 4–6 hours post-retrieval through either standard IVF or ICSI, depending on semen quality. Embryos graded 1–3 were selected for blastocyst development or cryopreservation 72 hours after retrieval, with vitrification occurring 120 hours post-retrieval upon blastocyst formation.

### FET endometrial preparation and FET

The study included 869 frozen embryo transfer cycles involving a total of 710 participants. Our two common endometrial preparation protocols involve hormone replacement therapy (HRT) alone or downregulation combined with HRT in subsequent cycles after oocyte retrieval (17). HRT protocol: women took 2 mg oral estradiol valerate tablets (Progynova, Berlin, Germany) twice daily from day 3 of spontaneous menses or progesterone-induced withdrawal bleeding. The dosage of Progynova was adjusted based on endometrial thickness and serum E2 levels, up to a maximum of 8 mg daily. After 16 days, once the endometrial thickness reached at least 7 mm and serum E2 levels were at least 100 pg/ml, luteal-phase support was initiated with 90 mg vaginal progesterone gel (Crinone; Merck Serono) or 60 mg intramuscular progesterone (Zhejiang Xianju Pharmaceutical Co., China) and 10 mg oral dydrogesterone twice daily. In the downregulation combined with HRT protocol, patients received a single intramuscular injection of 3.75 mg long-acting triptorelin acetate (Decapeptyl; Ferring, SaintPrex, Switzerland) on day 3 of the cycle. After 35 days of downregulation, oral estradiol valerate tablets were introduced, following the same procedure as the HRT protocol.

### Outcome

The primary end point was the cumulative live birth rate (cLBR), characterized as the count of deliveries resulting in at least one live birth from a full cycle of ART commencement, encompassing all FET cycles, until the attainment of a live birth or the utilization of all embryos, whichever occurred first. The secondary outcomes encompassed the occurrence of a premature surge in luteinizing hormone (LH), the count of oocytes retrieved, viable embryos, high-quality embryos, and high-quality blastocysts, as well as the clinical pregnancy rate, abortion rate, and ectopic pregnancy rate. The LH surge criteria were defined as serum LH levels exceeding 15.0 mIU/ml on the trigger day. Clinical pregnancy was determined by the presence of at least one fetus with discernible heart activity, assessed at least 30 days post-FET. Pre-specified safety endpoints included the percentage of women experiencing moderate or severe ovarian hyperstimulation syndrome (OHSS). Clinical manifestations such as ascites, pleural effusion, or dyspnea (whether exertional or at rest) are diagnostic criteria for moderate/severe ovarian hyperstimulation syndrome (OHSS) in women who exhibit more than one of these symptoms. The criterion for cycle cancellation was the absence of viable embryos suitable for cryopreservation. The cumulative live birth rate (cLBR) was computed as the ratio of the number of cycles resulting in live births to the total number of cycles of oocyte retrieval.

### Hormone analysis

Serum levels of FSH, LH, E_2_, and progesterone were assessed on the third day of the stimulation cycle (initiation of stimulation) and the day subsequent to hCG trigger (approximately 12 hours following the administration of GnRH-a and hCG). Electrochemiluminescence (Beckman Coulter, USA) was employed to measure hormone levels, with all assessments conducted by trained technicians adhering to the manufacturer’s guidelines. The sensitivity detection limits were as follows: FSH, 0.2 IU/L; LH, 0.2 IU/L; E_2_, 15 pg/ml; and P, 0.1 ng/ml. The in-house inter- and intra-assay coefficients of variation did not exceed 10%.

### Statistical methods

Data are presented as the mean ± standard deviation (SD) or as the frequency and percentage (for categorical variables). Continuous variables were expressed as either mean with standard deviation or median with interquartile range, and differences among groups were compared using one-way analysis of variance or the Kruskal-Wallis test. Categorical variables were presented as numbers with percentages and compared using Pearson’s chi-square test or Fisher’s exact test. Multivariable logistic regression analysis was conducted to evaluate significant associations between different dosage groups and pregnancy outcomes. Variables showing significance in the univariate analysis at a threshold of P<0.10 or higher, along with those potentially impacting live birth, were incorporated into the multivariable model. To pinpoint confounding factors influencing outcome measures, we initially conducted univariate analysis to assess the relationship between each covariate and individual outcome measures. Subsequently, in the basic model, covariates exhibiting statistical differences (p < 0.05) were introduced. In the complete model, covariates were systematically removed to observe changes in the regression coefficients of the covariates. Covariates demonstrating a change in regression coefficient exceeding 10% compared to the initial coefficient were deemed confounding factors and were subsequently adjusted for in subsequent multiple regression analyses. This methodology was employed to identify confounding factors influencing the number of retrieved oocytes, available embryos, high-quality embryos, clinical pregnancy, and cumulative live birth.

Through univariate analysis, we identified potential factors influencing the number of retrieved oocytes, embryonic characteristics, and pregnancy outcomes. Age, AMH, and AFC were recognized as confounding factors influencing the number of retrieved oocytes concerning daily MPA concentration. Age, AMH, AFC, embryo transfer stage, and the number of retrieved oocytes were recognized as confounding factors influencing the number of available embryos and high-quality embryos concerning daily MPA dosage. Likewise, age, AMH, AFC, infertility type, previous pregnancy history, number of retrieved oocytes, number of available embryos, number of high-quality embryos, and number of transferred embryos were deemed confounding factors impacting the influence of daily average MPA concentration on clinical pregnancy rate. Additionally, age, AMH, AFC, infertility type, previous pregnancy history, number of retrieved oocytes, number of available embryos, number of high-quality embryos were deemed confounding factors impacting the influence of daily average MPA concentration on cumulative live birth rate. Subsequently, we performed multivariate regression analysis to alleviate the impact of these confounding factors and explore the independent effects of daily average MPA dosage on clinical pregnancy rate and cumulative live birth rate.

The statistical packages R (The R Foundation; http://www.r-project.org;version 3.6.1), EmpowerStats (http://www.empowerstats.com) and SPSS 27.0 (IBM, Armonk, NY, USA) were utilized for all analyses.

## Result

### Patient characteristics

**Figure 1:**
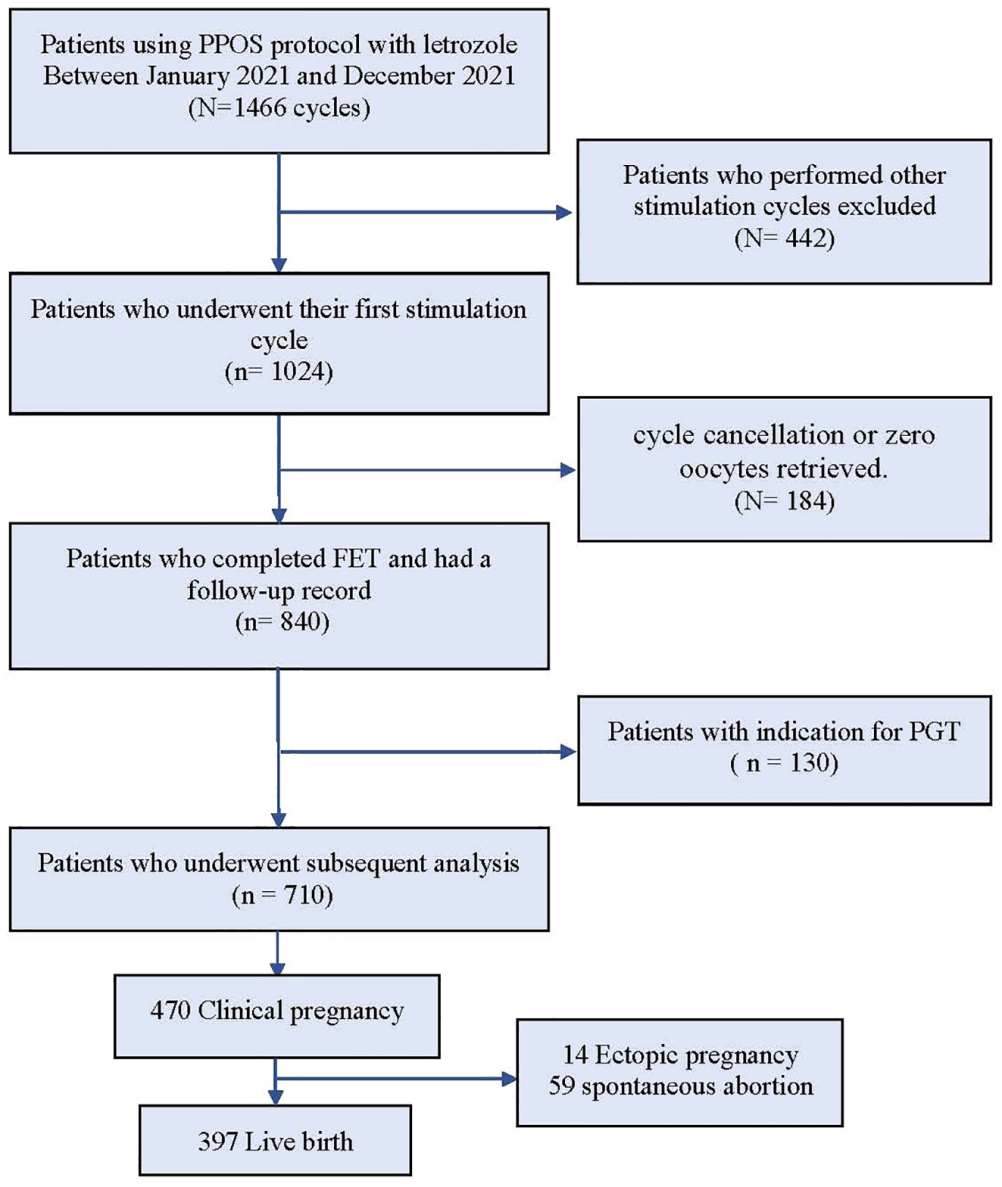
Flow chart for this trial.

The data selection process is illustrated in **Figure 1**. A total of 1466 IVF/ICSI cycles were extracted from our database, and among them, 1024 patients who exclusively underwent the first cycle with the PPOS protocol cotreatment with letrozole were chosen for analysis. 314 patients were excluded as described in the materials and methods section. The remaining 710 patients were categorized into three groups based on their daily average MPA dosage: high (6.5–10.0 mg), medium (4.7–6.4 mg), and low (1.0–4.6 mg). The three groups of patients showed significant statistical differences (p < 0.05) in BMI, history of pregnancy, infertility type, AMH, basal LH and AFC (**Table 1**).

**Table 1:** The basic characteristics of women in the trial undergoing IVF/ICSI treatment.

	Low group	Middle group	High group	P
Le PPOS Treatment, n	232	238	240	
Age (years)	33.3 ± 4.5	33.2 ± 5.0	32.7 ± 4.8	0.353
BMI (KG/m^2^)	24.4 ± 3.6	23.7 ± 3.3	22.5 ± 3.5	<0.001
Duration of infertility (years)	3.5 ± 2.7	3.6 ± 3.2	3.8 ± 3.3	0.539
Previous pregnancy				0.027
No	78 (33.6%)	89 (37.4%)	109 (45.4%)	
Yes	154 (66.4%)	149 (62.6%)	131 (54.6%)	
Types of infertility				0.023
Primary infertility	145 (62.5%)	136 (57.1%)	120 (50.0%)	
Secondary infertility	87 (37.5%)	102 (42.9%)	120 (50.0%)	
AMH (ng/ml)	2.5 ± 1.9	3.4 ± 3.2	4.7 ± 4.9	<0.001
Basal FSH (mlU/mL)	6.7 ± 3.2	6.8 ± 2.7	6.8 ± 3.3	0.872
Basal LH (mlU/mL)	4.3 ± 2.3	5.0 ± 2.0	6.1 ± 2.5	<0.001
Basal P (ng/mL)	0.5 ± 0.3	0.5 ± 0.3	0.6 ± 0.3	0.102
Basal E_2_ (pg/mL)	65.1 ± 76.5	68.9 ± 75.9	79.0 ± 73.6	0.069
AFC	8.6 ± 5.5	10.1 ± 7.3	12.0 ± 9.4	<0.001
Insemination method, n (%)				0.430
IVF	202 (87.1%)	204 (85.7%)	199 (82.9%)	
ICSI	30 (12.9%)	34 (14.3%)	41 (17.1%)	

### Ovarian stimulation characteristic

The ovarian stimulation characteristics of the three groups are given in **Table 2**. A total of 710 patients in three groups finished ovarian stimulation and oocyte retrievals. On the first day of ovarian stimulation, there was a significant difference in serum LH levels, with the high-dose group being significantly higher than the medium and low-dose groups (p < 0.05). Subsequently, the MPA dosage was adjusted based on the changes in patient serum LH levels.

**Table 2:** The controlled ovarian stimulation cycle characteristics.

	Low group	Middle group	High group	P
Le PPOS Treatment, n	232	238	240	
Trigger day				
LH (mIU/mL)	2.8 ± 1.7	3.2 ± 1.8	4.0 ± 2.0	<0.001
P (mIU/mL)	0.7 ± 0.4	0.8 ± 0.5	0.9 ± 0.5	0.033
E_2_ (pg/mL)	1990.5 ± 1184.3	2303.1 ± 1416.0	2823.6 ± 1640.6	<0.001
Premature LH surge	0	0	0	
Moderate/severe OHSS, n (%)	0	0	0	
No. of oocytes retrieved	6.9 ± 2.8	7.4 ± 3.1	8.1 ± 3.8	<0.001
No. of viable embryos	3.0 ± 1.3	3.2 ± 1.3	3.4 ± 1.4	0.011
No. of high-quality embryos	2.2 ± 1.3	2.4 ± 1.4	2.6 ± 1.6	0.022

On the trigger day, the LH levels, progesterone levels and Estrogen levels in the high-dose group were markedly higher than those in the low and medium-dose groups (P < 0.05). The high-dose group exhibited significantly higher numbers of retrieved oocytes, available embryos, and high-quality embryos compared to the medium and low-dose groups (P < 0.05). The confounding factors affecting the number of oocytes retrieval, the number of available embryos, and the number of high-quality embryos were determined by covariate screening, and the influence of confounding factors was eliminated by multiple regression analysis. After multivariate regression analysis, there were no significant statistical differences in the results among the three groups, and the regression coefficients did not exhibit significant changes.

### Hormone profile during treatment

**Table 2** displays the serum LH and progesterone levels on trigger day. The serum LH levels on the first day of stimulation showed a significant difference among the low, medium, and high-dose groups (low group: 4.3 ± 2.3, medium group: 5.0 ± 2.0, high group: 6.1 ± 2.5, p < 0.05). The serum LH levels on trigger day in each group were significantly lower than those on the first day of stimulation, but there were still significant statistical differences between the groups (low group: 2.8 ± 1.7, medium group: 3.2 ± 1.8, high group: 4.0 ± 2.0, p < 0.05). During ovarian stimulation, LH values gradually decreased. And no patients had premature LH surge or premature ovulation in any of the three groups. Additionally, no patients experienced moderate or severe OHSS during the study.

### Pregnancy outcomes in FET cycles

A total of 710 patients from the three groups completed 869 FET cycles. Descriptive statistic for the reproductive outcomes of FET are summarized (**Table 3**). The endometrial preparation protocols and thickness were similar in all three groups. The number of embryos transferred was also comparable across the three groups. To address potential confounding factors influencing pregnancy outcomes, a multivariate regression analysis was conducted on data related to pregnancy outcomes. Adjusting factors included age, AMH, AFC, infertility types, previous pregnancy history, number of embryos transferred, oocytes retrieved, available embryos and high-quality embryos. The daily dosage of MPA and the grouping based on the daily dosage of MPA served as the exposure variable, with clinical pregnancy rate and cumulative live birth rate investigated as outcome variables.

**Table 3:** FET characteristics and pregnancy outcomes after FET.

	Low group	Middle group	High group	P
Endometrium preparation, n (%)				0.447
De + HRT	199 (85.8%)	213 (89.5%)	208 (86.7%)	
HRT	33 (14.2%)	25 (10.5%)	32 (13.3%)	
Endometrial thickness (mm)	10.1 ± 2.1	9.9 ± 2.1	10.1 ± 2.0	0.409
Embryo transfer, n (%)				0.100
Cleavage stage	14 (6.0%)	8 (3.4%)	19 (7.9%)	
Blastocyst stage	218 (94.0%)	230 (96.6%)	221 (92.1%)	
No. of embryos transferred				0.364
1	92 (39.7%)	105 (44.1%)	91 (37.9%)	
2	140 (60.3%)	133 (55.9%)	149 (62.1%)	
Biochemistry pregnancy	69.4% (161/232)	72.3% (172/238)	71.7% (172/240)	0.770
Implantation rate	50.8% (189/372)	55.3% (205/371)	55.0% (214/389)	0.374
Clinical pregnancy rate	63.8% (148/232)	67.7% (161/238)	67.1% (161/240)	0.635
Multiple pregnancy rate	22.3% (33/148)	25.5% (41/161)	28.0% (45/161)	0.520
Ectopic pregnancy rate	3.4% (5/148)	3.1% (5/161)	2.5% (4/161)	0.893
Abortion rate	13.5% (20/148)	15.5% (25/161)	8.7% (14/161)	0.165
Cumulative live birth rate	53.0% (123/232)	55.0% (131/238)	59.6% (143/240)	0.337

Following the multivariate regression analysis, no significant differences were observed in pregnancy outcome indicators (**Table 4**). Before adjustment, a 1mg increase in the daily average MPA dosage corresponded to a 3% increase in the clinical pregnancy rate and a 6% increase in the cumulative live birth rate. However, after adjustment, each 1 mg increase in the daily average MPA dosage resulted in no change in the clinical pregnancy rate, and a 4% increase in the cumulative live birth rate. In comparison to the low-dose group, the clinical pregnancy rate increased by 17% in the medium-dose group and decreased by 1% in the high-dose group. Additionally, the cumulative live birth rate increased by 2% in the medium-dose group and increased by 12% in the high-dose group relative to the low-dose group.

**Table 4:** Stimulation and Pregnancy outcomes after Multiple regression analysis.

Exposure	Non-adjusted, β (95%CI)	Adjust I, β (95%CI)	Adjust II, β (95%CI)
No. of oocytes retrieved			
Dose of MPA per day	0.28 (0.15, 0.40) <0.0001	0.05 (-0.04, 0.15) 0.2856	-0.02 (-0.11, 0.07) 0.6462
Groups			
Low	0	0	0
Middle	0.48 (-0.11, 1.07) 0.1141	0.09 (-0.36, 0.53) 0.6974	-0.02 (-0.45, 0.41) 0.9345
High	1.22 (0.63, 1.81) <0.0001	0.30 (-0.15, 0.75) 0.1990	-0.01 (-0.45, 0.43) 0.9735
No. of viable embryos			
Dose of MPA per day	0.09 (0.04, 0.14) 0.0007	0.02 (-0.02, 0.06) 0.3925	0.01 (-0.02, 0.05) 0.4711
Groups			
Low	0	0	0
Middle	0.18 (-0.06, 0.42) 0.1503	0.06 (-0.15, 0.27) 0.5684	0.01 (-0.15, 0.17) 0.8693
High	0.37 (0.13, 0.61) 0.0028	0.08 (-0.12, 0.29) 0.4293	0.02 (-0.14, 0.19) 0.7853
No. of high-quality embryos			
Dose of MPA per day	0.08 (0.02, 0.13) 0.0045	0.02 (-0.03, 0.07) 0.5395	0.01 (-0.03, 0.06) 0.5118
Groups			
Low	0	0	0
Middle	0.18 (-0.07, 0.44) 0.1622	0.08 (-0.16, 0.31) 0.5189	0.04 (-0.17, 0.24) 0.7209
High	0.36 (0.11, 0.62) 0.0056	0.10 (-0.13, 0.34) 0.3857	0.07 (-0.14, 0.28) 0.5099
Clinical pregnancy rate per patient			
Dose of MPA per day	1.03 (0.95, 1.12) 0.4368	1.00 (0.92, 1.09) 0.9826	1.00 (0.91, 1.09) 0.9810
Groups			
Low	1.0	1.0	1.0
Middle	1.19 (0.81, 1.74) 0.3789	1.18 (0.78, 1.76) 0.4313	1.17 (0.78, 1.76) 0.4403
High	1.16 (0.79, 1.69) 0.4524	1.00 (0.67, 1.50) 0.9948	0.99 (0.66, 1.50) 0.9657
Cumulative live birth rate per patient			
Dose of MPA per day	1.06 (0.99, 1.15) 0.1108	1.04 (0.95, 1.13) 0.4245	1.04 (0.95, 1.13) 0.4218
Groups			
Low	1.0	1.0	1.0
Middle	1.08 (0.75, 1.56) 0.6597	1.02 (0.69, 1.51) 0.9066	1.02 (0.69, 1.51) 0.9042
High	1.31 (0.91, 1.88) 0.1508	1.12 (0.75, 1.67) 0.5934	1.12 (0.75, 1.68) 0.5810

## Discussion

A significant challenge in assisted reproduction is controlling premature ovulation. Recently, more flexible protocols have been introduced due to advancements in vitrification technology. For instance, progesterone has been widely used as an alternative to GnRH analogs to prevent premature ovulation (3, 18, 19). Among the various ovarian stimulation protocols, PPOS has been considered one of the key protocols (1, 20). The use of either endogenous progesterone or exogenous progesterone can effectively prevent LH surges during ovarian stimulation (20). Additionally, the use of progesterone can better control LH concentration, reduce costs, and facilitate (oral intake) administration (21). But the issues we can’t ignore are that progesterone levels before oocyte maturation may potentially damage oocytes. Additionally, some data suggest that elevated progesterone levels are associated with a lower rate of high-quality embryos (22, 23). In our study, there were no significant differences in the number of oocytes retrievals, the number of available embryos, and the number of high-quality embryos among patients with different daily MPA dosages. Meanwhile, a series of retrospective studies have shown reassuring neonatal outcomes following the use of MPA during ovarian stimulation (24, 25). Studies have also indicated that the PPOS protocol shows no significant difference in the safety of live birth outcomes compared to traditional ovarian stimulation protocols (26, 27). In accordance with these studies, our study showed that MPA at doses below 10 mg per day for approximately 10 days appeared to have a relatively respectable effect on embryo quality and pregnancy outcomes. Letrozole is a third-generation aromatase inhibitor that enhances ovarian sensitivity to Gn by increasing local ovarian androgen levels, the number of FSH receptors on granulosa cells, and the expression of insulin-like growth factors. Additionally, it promotes follicular development by inhibiting E_2_ synthesis, thereby stimulating the release of endogenous gonadotropins (28, 29). In our previous explorations,we reduced the use of MPA and exogenous Gn by adding a small amount of letrozole for a short period in the PPOS protocol (16, 30). Our prior investigation (16) has substantiated that incorporating letrozole into the PPOS protocol yields comparable embryo and pregnancy outcomes when juxtaposed with utilizing the PPOS protocol in isolation. Simultaneously, it diminishes the dosage of MPA and the quantity of drugs employed for ovulation stimulation, rendering it a more economically viable approach. In our study, all participants utilized the combined letrozole and PPOS protocol. During the ovarian stimulation process, the dosage of MPA was adjusted based on the changes in patient LH values. Patients were categorized into three groups—low, medium, and high concentration—based on the daily MPA dosage.

Recent research with varied MPA doses, such as 4 mg, 6 mg, and 10 mg daily, proved effective in preventing premature LH surges (4, 15, 31). A study indicated that infertile women using two different doses of MPA (10 mg and 4 mg daily) in the PPOS protocol had similar pregnancy outcomes and endocrine characteristics. In our cohort study, comparable findings were achieved and there were no premature LH peaks and excessively low LH values in the three groups. We further explored the embryonic characteristics and pregnancy outcomes of patients in three different MPA concentration ranges. Following adjustments for confounding factors, we assessed the independent impact of daily average MPA concentration on embryo characteristics and pregnancy outcomes. Across the three groups, number of retrieved oocytes, embryo characteristics (available embryos, high-quality embryos) and pregnancy outcomes (clinical pregnancy rate, cumulative live birth rate) displayed similarity, with no statistically significant differences. Furthermore, the comparable clinical pregnancy rates and cumulative live birth rates among the three groups imply that the combined letrozole with the PPOS protocol demonstrates embryo development potential and is minimally influenced by variations in daily MPA dosages.

While live birth is typically the gold standard for evaluating various treatments, it spans two cycles and involves additional confounding factors in the frozen-all strategy. Consequently, we selected the cumulative live birth rate as the primary measure for pregnancy outcomes. The primary objective of this trial is not only to discern the long-term effects of distinct MPA dosages on patients but also to unveil subtle changes directly indicative of the ovarian stimulation effects of varying MPA dosages. Hence, the number of retrieved oocytes is considered as a secondary outcome measure. An additional strength of this study lies in the meticulous control of LH levels. No occurrences of early LH peaks (LH > 15.0 mIU/ml on the trigger day) or LH suppression (LH < 1 mIU/ml on the trigger day) were noted throughout the ovarian stimulation process. Baseline LH values for the three groups were as follows: low group (4.4 ± 2.2), medium group (5.0 ± 1.9), high group (6.0 ± 2.4). Similarly, LH values on the trigger day were: low group (2.9 ± 1.7), medium group (3.2 ± 1.8), high group (4.0 ± 1.9). The comparable degree of change in LH values suggests a similar inhibitory effect on the pituitary gland across all three groups. Finally, to prevent underestimation of the actual cLBR, we routinely employed embryo vitrification for all patients involved in the study. We conducted a two-year follow-up for all patients, continuing until the first live birth or until all surplus frozen embryos were used.

However, we need to cautiously address the limitations present in this study that need to be emphasized. Firstly, the retrospective study design is inherently associated with biases that could potentially affect our results. Although, as compared with other cohorts, we attempted to minimize temporal bias by exclusively using data from the year 2021, avoiding historical controls, and mitigating confounding bias by controlling for a large number of known confounding factors. However, we cannot entirely rule out the presence of some unknown confounding factors. Furthermore, our cohort consisted of relatively young patients with good prognoses who were undergoing their first ovarian stimulation cycle. Therefore, caution is needed when applying our results to less favorable IVF populations.

Another constraint is that the modest sample size could undermine the generalizability of the results. Moreover, the precise minimum effective dose of MPA remains unclear. At our center, the initial progestin dose for inducing ovulation in the PPOS protocol is MPA 8–10 mg/day, with gradual adjustments based on serum LH levels. Despite the low concentration group in our study having a range of 1 mg to 4.6 mg, with a minimum value of 1 mg, our discussion pertains to a broad concentration range rather than specific numerical values. Consequently, multicenter large-sample trials are essential to further validate a more precise range. Additionally, it’s important to note that this study is retrospective rather than prospective.

## Conclusion

The study indicated that PPOS using different doses of MPA per day produced similar endocrinological characteristics and pregnancy outcomes in infertile woman undergoing control ovarian hyperstimulation. Furthermore, there was no early LH peak observed within the studied range of MPA concentrations. Therefore, we deduce that when the concentration of MPA is low to a certain extent, it can both prevent early LH peaks and not impede the synchronized development of the endometrium, thereby facilitating fresh embryo transfer in the PPOS protocol.

## Data availability statement

The raw data supporting the conclusions of this article will be made available by the authors, without undue reservation.

## Ethics statement

This study protocol was formulated in accordance with the requirements of the Declaration of Helsinki of the World Medical Association. The studies involving humans were approved by Ethics Committee of Renmin Hospital, Hubei University of Medicine. The studies were conducted in accordance with the local legislation and institutional requirements. Informed consent forms were provided by all patients after they received counseling on infertility treatment and the routine ART process.

## Author contributions

H-LL: Data curation, Investigation, Methodology, Software, Writing – original draft, Writing – review & editing. B-BS: Data curation, Investigation, Methodology, Software, Writing – review & editing. Z-LH: Investigation, Validation, Writing – review & editing. H-LW: Data curation, Investigation, Writing – review & editing. ZS: Data curation, Funding acquisition, Investigation, Project administration, Resources, Software, Validation, Writing – original draft, Writing – review & editing.
